# Factors Associated with Nutritional Risk Assessment in Critically Ill Patients Using the Malnutrition Universal Screening Tool (MUST)

**DOI:** 10.3390/jcm13051236

**Published:** 2024-02-21

**Authors:** Blanca Cecilia Díaz Chavarro, Guillermo Molina-Recio, Jorge Karim Assis Reveiz, Manuel Romero-Saldaña

**Affiliations:** 1 Nursing Program, School of Health, Research Group Genetics, Physiology and Metabolism (GEFIME), Universidad Santiago de Cali, Santiago de Cali 760001, Colombia; blanca.diaz00@usc.edu.co; 2 Doctoral Program in Biosciences and Agricultural and Food Sciences, University of Córdoba, 14014 Cordoba, Spain; 3 Nursing, Pharmacology and Physiotherapy Department, University of Cordoba, 14004 Cordoba, Spain; z92rosam@uco.es; 4 Lifestyles, Innovation and Health (GA–16), Maimonides Biomedical Research Institute of Cordoba (IMIBIC), 14014 Cordoba, Spain; 5 Department of Research and Education, Clínica de Occidente SA, Santiago de Cali 760001, Colombia; ophthalmology2013@gmail.com

**Keywords:** nutritional status, nutritional assessment, critical patients, nutritional risk, mortality

## Abstract

**Background**: Malnutrition is an underdiagnosed condition that negatively affects the clinical outcomes of patients, being associated with an increased risk of adverse events, increased hospital stay, and higher mortality. Therefore, nutritional assessment is a required and necessary process in patient care. The objective of this study was to identify the factors associated with nutritional risk by applying the Malnutrition Universal Screening Tool (MUST) scale in a population of critically ill patients. **Methods**: This was an observational, analytical, and retrospective study. Sociodemographic, clinical, hematological, and biochemical variables and their relationship with nutritional risk and mortality were analyzed. **Results**: Of 630 patients, the leading cause of admission was pathologies of the circulatory and respiratory system (50%); 28.4% were at high nutritional risk; and mortality was 11.6% and associated with nutritional risk, hemoglobin, and plasma urea nitrogen. **Conclusions**: The presence of gastrointestinal symptoms and the type of nutritional support received during hospitalization could increase the likelihood of presenting a medium/high nutritional risk, while polycythemia reduced this probability. An associative model was found to determine nutritional risk with an adequate specificity and diagnostic validity index.

## 1. Introduction

Malnutrition, characterized by deficiencies in energy, protein, and essential nutrients [1], profoundly affects bodily functions [2]. This problem is widespread in hospital settings worldwide, spanning continents such as Europe [3], Australia [4,5], Asia [6], North America [7], and Latin America [8], and its consequences occur more rapidly and are more evident in critically ill patients [9]. Additionally, it remains an underdiagnosed condition [10], leading to inadequate treatment, often associated with healthcare professionals’ insufficient knowledge and inadequate clinical approaches and practices [11]. Clinical behaviors contributing to this issue encompass food intake interruption for diagnostic or therapeutic purposes, time constraints impeding nutrition-focused care, and absence of intervention in cases of inadequate patient food intake [12,13].

Malnutrition has a high frequency in the Intensive Care Unit (ICU) [9] and significantly compromises clinical outcomes [1], impacting tissue metabolism, muscular strength, wound healing, and immune function [2]. It is associated with a higher risk of adverse events including infectious and noninfectious complications, prolonged need for mechanical ventilation, longer rehabilitation process [14,15,16], most frequent readmission to the ICU [8], extended hospital stay, and heightened mortality risk [17]. Therefore, it increases healthcare expenses, compromises patients’ quality of life, and imposes additional financial burdens on healthcare institutions [6,18].

Thus, routine nutritional assessments and monitoring play a crucial role in patient care by enabling the identification of deficiencies via screening, evaluating, and diagnosing the nutritional status [8]. This practice aids in predicting positive or negative health outcomes [19]. It is integrated as a fundamental part of managing hospitalized ICU patients and as a therapeutic strategy during their care [20]. For critically ill patients, oral food intake is frequently impaired [21], so feeding protocols are required for adequate nutritional status and risk reduction during hospitalization [22].

A literature review shows that compliance with the guidelines for the nutritional management of critically ill patients is poor [23]. There is a lack of clinical records on nutritional variables. In addition to this, the clinical condition of patients requires the use of resuscitation maneuvers and the administration of a large volume of fluids [24]. Therefore, assessing isolated weight changes may lack nutritional significance owing to confounding factors related to patients’ hydration status [10].

The prevalence of malnutrition in the ICU is between 33% and 78% [9,15,25,26,27], showing a higher likelihood of malnutrition than patients hospitalized in general services [28]. As critically ill patients are in an increased proinflammatory state [29], the effects of malnutrition may be intensified. Therefore, it is essential to assess its presence and related factors [27]. For these reasons, multiple nutritional assessment scales are currently available; however, none hold the status of being the definitive “gold standard” for critically ill patients [24]; consequently, each institution opts for the one that best aligns with the characteristics of its patient population [30]. Among these methodologies, the Malnutrition Universal Screening Tool (MUST) emerges as a straightforward assessment tool designed to identify adult patients with malnutrition in all healthcare settings who require subsequent monitoring and nutritional intervention [31].

Lew et al. conducted a systematic review and compared the use of different nutritional screening and assessment tools in the ICU, including the Nutrition Risk Screening-2002 (NRS), the Malnutrition Universal Screening Tool (MUST), the Subjective Global Assessment (SGA), and the Mini Nutritional Assessment (MNA), among others. It was found that the NRS-2002 and the MUST had a better predictive value among the nutritional screening tools compared. The authors indicate that these similarities could be related to the data collected in both instruments, which include the body mass index (BMI) and recent weight loss [27].

Considering these factors, the primary objective of this study is to identify the factors associated with nutritional risk by employing the MUST scale in a cohort of critically ill patients from a hospital in Cali (Colombia).

## 2. Materials and Methods

### 2.1. Design, Population, and Sample

An observational, analytical, and retrospective study was conducted with adult patients hospitalized in the ICU of a level IV clinic located in the city of Cali (Colombia). The study spanned from 1 January 2021 to 30 April 2022.

The ICU is distributed in 6 modules for critically ill patients, both for medical and surgical causes. Thus, respiratory, cardiovascular, surgical, neurovascular, general, and intermediate care ICUs exist.

**Figure 1:**
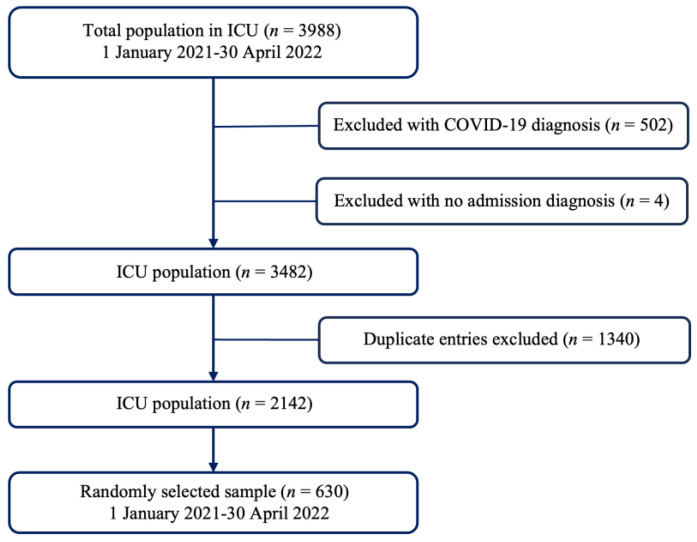
Sample selection flow diagram.

The population encompassed a total of 3988 admitted patients during this period, 2142 of which were considered potential candidates for inclusion in the study (Figure 1).

For sample size estimation, considering the risk of malnutrition ranging from 40% to 60% in hospitalized Latin American patients [32], and aiming for a 95% confidence level with an estimation precision of 4%, a minimum sample size of 454 patients was calculated. Ultimately, a study sample of 630 individuals was selected through a simple random sampling technique stratified by age and sex using the Epidat tool ver. 4.2.

### 2.2. Eligibility Criteria

The study included adult patients admitted to the ICU during the investigation period who underwent nutritional risk assessment using the MUST scale.

Patients diagnosed with COVID-19 were excluded due to the distinct impact on nutritional status attributed to gastrointestinal and immune system disruptions, escalated metabolic activity due to the infectious process, presence of fever, and reduced oral food intake [33]. Furthermore, individuals lacking medical diagnosis data upon admission to the ICU or those with duplicate records generated within the hospital’s system due to patient transfers within the institution’s services, be it for diagnostic or surgical procedures, were excluded (Figure 1).

### 2.3. Variables and Measurement

#### 2.3.1. Explanatory Variables

Sociodemographic variables such as sex, age, and admission disease according to ICD 11; hematological variables such as hemoglobin (normal range: 12.3–15.3 g/dL), leukocytes (normal range: 4.5–11 × 10^3^/μL) and lymphocyte levels (normal range: 1.0–4.8 × 10^3^/μL); and biochemical variables, such as urea nitrogen (normal range: 7–20 mg/dL), creatinine (normal range: 0.7–1.4 mg/dL), potassium (normal range: 3.5–5.0 mEq/L), and sodium levels (normal range: 135–145 mEq/L), were considered (Table S1).

Furthermore, the presence of gastrointestinal symptoms (hyporexia, abdominal distension, diarrhea, swallowing difficulties, emesis, and abdominal pain) and the type of nutritional support received (oral, enteral, and parenteral) were included.

#### 2.3.2. Outcome Variable

As outcome variable, nutritional risk assessed using the MUST scale—categorized into low, medium, or high risk—and vital status at discharge (alive or deceased) were considered.

### 2.4. Assessment of Nutritional Needs and Care

Those responsible for the MUST scale assessment were the nutritionists of the health institution in response to the request of the ICU medical team. The frequency of the assessment depended on the level of risk identified in each patient. In the case of low-risk patients, it was established that the assigned physician would establish the dietary guidelines, and the screening was repeated weekly. For medium-risk patients, the dietary intake was monitored for three consecutive days, and if it was sufficient, a new screening was carried out every week. For patients at high nutritional risk, follow-up was performed by the nutrition and dietetics unit, which established a treatment and assessment protocol and reviewed the nutritional care plan.

Gastrointestinal symptoms were reported by both the medical staff and the nutrition and dietetics team during the patient’s admission assessment and nutritional screening. This information was obtained through the anamnesis with verbal reference from the patient (if the patient’s clinical condition permitted) or information provided by the patient’s primary caregiver. In addition, this information was completed with data from the physical assessment performed by the physician in charge of the patient in the ICU. In the case of hyporexia, it was documented based on the verbal reference of the patient’s decreased appetite and decreased oral intake.

The nutritional support collected in the study corresponds to that indicated in the patient’s clinical history, according to the MUST tool assessment at the time of interconsultation with the nutrition and dietetics team. This study did not record changes in the type of nutritional support, considering that only the data from the first nutritional screening were collected, together with the results of the health workers who transferred the patient on admission. The reason was to have the information to analyze the patient’s condition at a single moment of hospitalization and that all the data were related to the nutritional risk identified according to the MUST.

### 2.5. Measurement Instruments

The MUST scale was employed to assess the risk of malnutrition. This tool demonstrates a sensitivity of 80.2% and exhibits a high discriminative capability (area under the ROC curve of 0.868) in determining nutritional risk. This evaluation was compared against a comprehensive nutritional assessment that includes participants’ medical history, dietary-nutritional history, pharmacological treatment, physical examination, anthropometric measurements, and laboratory data [34].

The MUST scale considers the analysis of three scores: (a) Body mass index (BMI) calculated as kg/m^2^, which is scored ≥20 kg/m^2^ = 0; 18.5–20 kg/m^2^ = 1; and ≤18.5 kg/m^2^ = 2. (b) Unintentional weight loss (WL) during the last 3–6 months, calculated as a percentage and coded as follows: WL ≤5% = 0; WL 5%–10% = 1; and WL ≥10% = 2. (c) The effect of acute diseases, in which 2 points are assigned when there has been or is likely to be no nutritional intake for >5 days [35,36].

The cumulative value for these three scores categorizes patients into three risk levels: 0 points = low risk; 1 point = medium risk; ≥2 points = high risk [35,36].

### 2.6. Ethical Considerations

This research adhered to the ethical guidelines of Council for International Organizations of Medical Sciences and the Declaration of Helsinki regarding the participation of human beings, including the signing of an informed consent prior to the collection of information, either by the patient or the responsible family member. The Research Ethics Committee of the Faculty of Health of the Universidad Santiago de Cali, Colombia, and the Scientific Technical Committee of the participating Clinic (Record IYECDO–1358) approved the study.

### 2.7. Statistical Analysis

Statistical analyses were conducted using SPSS software version 28.0. Quantitative variables were represented using arithmetic mean and standard deviation values. The Kolmogorov–Smirnov test with Lilliefors correction was used to analyze the goodness of fit of the data at a normal distribution. Mean variation was calculated through the application of ANOVA for one factor, considering a value of *p* < 0.05 as significant. For the comparison of nominal variables, Pearson’s Chi-square test was used with Fisher’s exact test when required.

Moreover, binary logistic regression was performed to analyze nutritional risk, categorized into low and medium/high. An initial crude analysis was performed to identify the associated variables, and subsequently, the adjusted estimation was performed, estimating OR values for the determination of risk and evaluating the goodness of fit of the model through the determination of Cox and Snell’s R^2^, Nagelkerke’s R^2^, and Hosmer and Lemeshow’s test. Finally, the validity index, the correct percentage of patient classification, the area under the curve (ROC), and the diagnostic accuracy of the variables proposed in the model for the determination of nutritional risk were determined, and comparisons were made with the results of the MUST scale as gold standard. This was performed by calculating the sensitivity, specificity, and positive and negative predictive value (PPV and NPV) of the model, as well as the Youden index.

## 3. Results

### 3.1. Characterization of the Study Participants

The study comprised a total of 630 patients, with 341 being male (54.1%). The overall mean age of the sample was 64.75 (SD = 16.21) with a 95% CI (63.49–66.02).

A high prevalence of circulatory and respiratory diseases (50%) was observed as a cause of admission to the ICU, with significantly higher occurrence among male patients (61%).

Male patients exhibited a higher average weight, recorded at 70.08 kg (±13.68). The MUST scale identified a nutritional risk of 28.4%, identifying a higher proportion of male patients categorized under low nutritional risk. Finally, the mortality rate among the study sample was 11.6% (Table 1).

### 3.2. Hematological and Biochemical Parameters Associated with Nutritional Risk

Regarding the laboratory tests conducted on the participants, an association between nutritional risk and hematological parameters (such as hemoglobin and hematocrit values) was identified, showing a lower average value (10.84 g/dL, 33.49% respectively) in patients with high nutritional risk. In the analysis of the white series, an average leukocyte count in patients of 10.68 × 10^3^/μL (SD = 17.22) 95% CI (9.33–12.03) was observed and, as in the biochemical tests such as renal function and electrolyte levels in blood plasma, no significant associations were identified according to nutritional risk (Table 2).

### 3.3. Morbidity, Gastrointestinal Symptoms, and Route of Nutritional Support Associated with Nutritional Risk

Table 3 shows other factors related to nutritional risk, including the diseases causing admission to the ICU, the presence of gastrointestinal symptoms, and the route of nutritional support received. The percentage of patients at low risk was higher, and diseases of the circulatory and respiratory system (67%) and those of the nervous system, musculoskeletal system and/or trauma (65.1%) stood out. The patients with the highest prevalence of high nutritional risk were those with infectious or parasitic diseases (42.4%). In the case of gastrointestinal symptoms, we found an association between the high nutritional risk of malnutrition and the presence of hyporexia, bloating, or abdominal pain; diarrhea; and swallowing difficulties or emesis, obtaining prevalence values in patients between 36.1% and 47.8%. The route of nutritional support with the greatest effect on the increase in nutritional risk was the parenteral one, with 77.3% of patients at high risk.

### 3.4. Factors Related to Mortality in the ICU

Patients with low nutritional risk exhibited a greater survival rate (62.1%). For hematological and biochemical parameters, an evident correlation emerged between hemoglobin and blood urea nitrogen (BUN) levels and vital status at discharge, showing lower hemoglobin values, with a mean of 10.39 g/dL (SD = 20.06) in deceased patients. Elevated BUN levels were also found in these patients, with a mean of 35.45 mg/dL (SD = 25.33) (Table 4).

### 3.5. Multivariate Analysis for Nutritional Risk

Variables associated with nutritional risk in patients hospitalized in the ICU were identified and included in the adjusted binary logistic regression model with the following outcome variables: low risk and medium/high risk. This adjusted model showed a validity index of 65.40%, specificity of 81.98%, PPV of 58.68%, and NPV of 67.82%.

In particular, patients with hyporexia were 1.82 times more likely to have a medium/high nutritional risk. In contrast, patients with polycythemia demonstrated a protective effect against this nutritional risk. In addition, it was observed that patients with parenteral nutritional support presented an OR of 5.61 compared to the oral route since parenteral nutrition is indicated in those critical patients with severe malnutrition. Therefore, this type of nutrition is expected to present this high OR value. It does not suggest that parenteral nutrition is a risk of malnutrition but, on the contrary, a therapeutic indicated by malnutrition. Considering the importance of this result, this variable was kept in the regression model; since her nutritional risk continues, being necessary a strict follow-up to determine her progress and the requirement of adjustments in nutritional care to reduce the complications associated with her metabolic and nutritional status (Table 5).

## 4. Discussion

Our study assessed the nutritional risk of 630 critically ill patients hospitalized in a health institution in Cali, Colombia, during 2021 and 2022 and its association with clinical variables (hematological and biochemical parameters, gastrointestinal symptoms, and type of nutritional support required in the ICU).

### 4.1. Diseases and Nutritional Risk by Sex

The average age varied between men and women across different studies. For instance, research on an ICU in Korea revealed a higher mean age among women at 67.8 years (SD = 15.8), compared to an average of 62.4 years (SD = 14.6) in male patients [37]. A similar trend was observed in a study conducted in New South Wales, Australia, with a mean age of 63.7 years in females compared to 61.8 years in males (*p* < 0.001) [38].

Regarding reasons for ICU admission, circulatory and respiratory diseases prevailed, which is consistent with a study conducted in the United States with a predominance of patients with respiratory failure (42%). However, the second most frequent cause was related to nonrespiratory sepsis (17%) [39], unlike the results of our study, in which the second most frequent disease was neoplasms.

Sociodemographic characterization in health research involves analyzing the behavior of events and categorizing them based on these variables. For example, one study found lower rates of heart disease in the female population, with 8% compared to 16% in men [38]. These results coincide with our population in Cali, Colombia, with rates of circulatory and respiratory diseases being 20% lower in women.

Endocrine and digestive diseases were more prevalent in female patients (59.6%), results that differ from those found in Korean ICUs, where there was a higher prevalence of liver disease in men [37]. In terms of infectious diseases, there was no significant difference between the number of male and female patients. This finding was also identified in the analysis of the data collected in Australia, with patients admitted to the ICU for sepsis [38].

When analyzing nutritional status by the BMI, an Australian study evidenced significant differences in terms of a higher proportion of a healthy BMI (>18.5 ≤25 kg/m^2^) in women (39.3%) than in men (29.0%) [38]. This was not observed in the results of our study and coincided with another research conducted in the USA [40]. Regarding patient mortality, the literature has reported 19.7% of the African population hospitalized in the ICU without a diagnosis of COVID-19 [41] and 19.1% of the population admitted to the medical and surgical ICU at Brigham and Women’s Hospital in Boston, Massachusetts [15]. These figures are lower than those reported in our results (11.6%).

### 4.2. Hemoglobin and Electrolyte Values Associated with Nutritional Risk

Hemoglobin levels serve as indicative biomarkers reflecting both nutritional status and the physiological stress associated with a patient’s disease [42]. Consequently, constant monitoring of this parameter remains pivotal [43], especially in critically ill patients due to the high incidence of anemia [44], which is associated with adverse outcomes in those with congestive heart failure [45], acute myocardial infarction [46], chronic kidney disease [47], and chronic obstructive pulmonary disease [48]. This situation is associated with failures in the withdrawal of mechanical ventilation of critically ill patients [49], increased risk of mortality [50], and the respective increase in the use of health care resources [51].

The presence of anemia in critically ill patients is the result of a shorter circulatory life of red blood cells and the decrease in their new production fundamentally related to nutritional deficiencies [51]. In addition, an increase in the hemoglobin levels of patients has been demonstrated after a higher intake of calories and proteins during hospitalization [52]. In this regard, our findings suggest that the lowest average hemoglobin level was present in patients at high nutritional risk (according to MUST), probably related to WL and low nutritional intake.

Conversely, the lymphocyte count has been included as a marker of immune competence and an indicator of the nutritional status of patients in the ICU [53]. This fact, reflected in the results of our work, was also observed in a study conducted in Korean critically ill patients, which evaluated changes in nutritional status according to the Subjective Global Assessment (SGA) and energetic intake, finding that total lymphocytes decreased only in the group of patients with severe malnutrition [54].

Furthermore, a lower lymphocyte count was also found in a population of cardiac surgery patients admitted to the ICU and subsequently hospitalized, who were classified according to the Geriatric Nutritional Risk Index (GNRI) as patients with increased nutritional risk. In addition, in cardiac surgery, patients hospitalized in the ICU were identified with decreased sodium levels and, in turn, higher serum potassium levels in the group of patients with higher nutritional risk [55]. Although these associations were not statistically significant in the current results, where the electrolytes analyzed were not associated with nutritional risk, the European Society for Clinical Nutrition and Metabolism (ESPEN) guidelines indicate that monitoring laboratory variables such as sodium and potassium levels in critically ill patients have been associated with clinical complications and poor health outcomes. Therefore, they should be considered part of the nutritional follow-up performed on patients [56].

### 4.3. Morbidity, Gastrointestinal Symptoms, and Nutritional Support Route Associated with Nutritional Risk

In 2021, the British Association for Parenteral and Enteral Nutrition reported that 40% of hospitalized adult patients were at risk of malnutrition, according to the MUST tool. Notably, the prevalence was notably high among patients with gastrointestinal diseases (55%), respiratory diseases (48%), and cancer (47%) [57]. These results coincide with our research, where the highest percentages of medium/high nutritional risk were found in patients with neoplastic (46.7%) and gastrointestinal (53.3%) diseases. In the case of cancer patients, malnutrition is increased by factors related to systemic inflammatory processes, metabolic disorders such as proteolysis and lipolysis, and factors associated with the disease or side effects of treatment, which generate decreased nutritional intake, lack of appetite, and abdominal distension [58]. These symptoms are also very frequent in patients with gastrointestinal diseases and are responsible for a greater deterioration of nutritional status among hospitalized patients [59].

Regarding gastrointestinal symptoms such as hyporexia, bloating, abdominal pain, diarrhea, swallowing difficulties, emesis, and their association with nutritional risk identified in this study, Pearcy et al. have indicated that critical illness includes these types of symptoms, with increased catabolism due to the inflammatory response and a frequent inability to ingest food orally, thus raising the patient’s nutritional needs. Consequently, feeding protocols that address factors that delay the use of enteral nutritional support, including gastrointestinal dysfunction, increase the provision of nutritional therapy and ensure greater nutrient delivery, decreasing nutritional risk [23].

Since these gastrointestinal symptoms reduce nutritional intake, generate malabsorption of food, and affect nutritional status, they have recently been introduced in the Global Leadership Initiative on Malnutrition (GLIM) etiological criteria for the analysis of the nutritional status of patients [60], including those in the ICU [30,61]. These criteria establish a two-step approach to diagnose malnutrition in health care settings, proposing a first step with nutritional risk screening through a valid instrument and subsequent investigation of phenotypic criteria such as WL, the BMI, reduced body mass, and etiological criteria such as reduced food intake, nutrient absorption, and presence of inflammation [62].

While parenteral nutrition is reserved for patients unable to receive nutrition orally or through enteral means, being a less frequent supplementary route [23], only 3.5% of the patients in our study received this type of nutritional support. It was also observed that the type of intervention was associated with the MUST risk identified, which may explain why 48.1% of the high-risk patients in our sample received enteral support. According to the analysis of NutritionDay data collected between 2006 and 2019 by Tarantino et al., the use of parenteral nutritional support in critically ill patients has varied in different European countries, ranging from 1% to 13%. These differences would be related to the interpretation of each country’s specific nutritional care guidelines [63].

According to the guidelines of the European Society for Clinical Nutrition and Metabolism (ESPEN), the Society of Critical Care Medicine (SCCM) and American Society for Parenteral and Enteral Nutrition (ASPEN) [21,23], enteral nutritional support should be performed early in critically ill patients without oral intake [21] with the aim of preventing further nutritional deterioration associated with the disease or treatment [57], thereby lowering mortality risks. As evidenced in the population of Cali, Colombia, patients with higher nutritional risk presented higher mortality at discharge. Therefore, and according to the complexity involved in defining the feeding route, a formal evaluation of risks and benefits with the patient and family seems necessary, where the perspectives of a multidisciplinary team allow the design of a nutritional care plan adapted to the identified needs [64].

### 4.4. Nutritional Risk and Hematological and Biochemical Parameters Associated with Mortality in the ICU

Patients with malnutrition exhibit an increased likelihood of experiencing adverse outcomes, including increased mortality risk during hospitalization and up to 6 months after discharge [60]. This was evident in the finding of the Modified Nutrition Risk in the Critically Ill score in patients admitted to the ICU of the Gangnam Severance Hospital in Seoul, South Korea. This work showed that the nutritional risk scores were significantly higher in the nonsurvivors group. In addition, they had elevated BUN levels, with an average of 34.6 mg/dL (SD = 24.3), compared to 19.3 mg/dL (SD = 19.1) in the survivors [53]. In this same study, statistically significant differences were also found in lymphocyte levels, with a value of 1322.2/μL (SD = 974.0) in survivors versus 1084.4/μL (SD = 1105.4) in nonsurvivors [53].

All these data are consistent with the findings of our study, affirming that those patients with lower nutritional risk had a higher percentage of survival, while deceased patients showed significantly higher values of BUN in blood plasma and lower lymphocyte counts.

### 4.5. MUST Nutritional Risk Associative Model

The work of Rattanachaiwong et al. compared the use of four tools for identifying patients with severe malnutrition in the ICU, including the Nutrition Risk Screening (NRS), the Nutrition Risk in Critically Ill (NUTRIC), the malnutrition criteria proposed by ESPEN and the criteria of the American Society for Parenteral and Enteral Nutrition (ASPEN), using the SGA as the gold standard. The NRS showed the highest sensitivity for identifying severe malnutrition at 79.07% and high specificity at 94.81%. NUTRIC, on the other hand, had the least effective performance in this diagnosis in the ICU, with a sensitivity of 58.14% and a specificity of 74.03% [65].

When comparing these findings with our associative model, which includes the presence of hyporexia, the interpretation of hemoglobin values, and the route of nutritional support used in the patient, we can also observe that sensitivity values (39.68%) are lower than specificity values (81.98%). This is an associative model that could be useful to identify hospitalized populations with low nutritional risk.

These diagnostic accuracy measures and their low sensitivity values might be associated with the exclusion of data on the BMI or involuntary WL in patients. This is because these were not considered as independent variables in the model since both are included in the outcome variable of the MUST scale, which would statistically imply collinearity between these variables. However, in comparing the abovementioned instruments, each has implicit data related to the BMI or WL [65]. Consequently, it seems evident that the inclusion of these variables increases the predictive capacity and complementarity between each scale and the SGA.

### 4.6. Study Limitations

One limitation of our study involved the impact of the COVID-19 pandemic, which posed challenges in sample collection due to the distinct pathophysiological features outlined in the selection criteria.

Moreover, the associative model of nutritional risk was established and assessed in a population with very specific sociodemographic and clinical characteristics, so it is necessary to validate the model in other populations to corroborate its diagnostic accuracy. In addition, it seems evident that there is a need to identify a variable that could increase the sensitivity of this model, which would improve its discriminant capacity and diagnostic validity in the identification of patients with medium/high nutritional risk.

## 5. Conclusions

Our study showed that the presence of a gastrointestinal symptom, such as hyporexia, has a direct effect on the increase in nutritional risk, as does the use of enteral or parenteral nutritional support as a substitute for oral intake. Conversely, polycythemia reduces the probability of showing medium/high nutritional risk by 38%.

Within this ICU patient sample, an associative model was found to determine nutritional risk based on the presence of hyporexia, the interpretation of hemoglobin levels, and the route of nutritional support used. This model has obtained a high specificity and an adequate index of diagnostic validity for the classification of patients with nutritional risk.

## Figures and Tables

**Table 1:** Characterization of patients admitted to the ICU.

Variables	$\mathrm{Total}\;\bar{x}$ (±SD) *n* (%)	$\mathrm{Female}\;\bar{x}$ (±SD) 289 (45.87%)	$\mathrm{Male}\;\bar{x}$ (±SD) 341 (54.13%)	*p*
Age	64.75 (±16.21)	63.32 (±18.08)	65.97 (±14.35)	0.041 *
Cause of admission ICD-11				
Circulatory and respiratory diseases	315 (50)	123 (39)	192 (61)	0.011 *
Neoplasms	107 (17)	50 (46.7)	57 (53.3)	
Nervous and musculoskeletal diseases and trauma	83 (13.1)	45 (54.2)	38 (45.8)	
Endocrine and digestive diseases	47 (7.5)	28 (59.6)	19 (40.4)	
Infectious or parasitic diseases	33 (5.2)	17 (51.5)	16 (48.5)	
Other diseases	45 (7.1)	26 (57.8)	19 (42.2)	
Days of hospitalization	6.06 (8.49)	6.25 (10.24)	5.90 (6.69)	0.612
Deaths	73 (11.6)	35 (48)	38 (52.)	0.706
Weight	66.35 (±13.78)	61.95 (±12.55)	70.08 (±13.68)	<0.001 *
BMI	24.76 (±4.75)	24.83 (±4.98)	24.70 (±4.57)	0.746
Nutritional risk				
Low risk	383 (60.8)	161 (42)	222 (58)	0.054
Medium risk	68 (10.8)	36 (52.9)	32 (47.1)	
High risk	179 (28.4)	92 (51.4)	87 (48.6)	

$\bar{x}$: Average; ±SD: Standard deviation; Statistical tests: One-way ANOVA and Pearson’s Chi-square; * Significant differences *p* < 0.05.

**Table 2:** Hematological and biochemical parameters associated with nutritional risk.

Laboratory Tests	$\bar{x}$ (±SD)	MUST Scale			
		Low Risk	Medium Risk	High Risk	*p*
Hemoglobin (g/dL)	11.46 (2.60)	11.79 (2.67)	11.21 (2.55)	10.84 (2.32)	<0.001 *
Hematocrit (%)	35.37 (7.68)	36.35 (7.80)	34.76 (7.91)	33.49 (6.98)	<0.001 *
Leukocytes (×10^3^/μL)	10.68 (17.22)	10.21 (11.05)	9.14 (5.82)	12.27 (27.73)	0.308
Lymphocytes (×10^3^/μL)	2.01 (9.77)	2.08 (10.86)	1.43 (0.70)	2.04 (8.89)	0.876
BUN (mg/dL)	25.63 (18.41)	25.40 (18.49)	23.72 (16.68)	26.83 (18.88)	0.460
Creatinine (mg/dL)	1.61 (2.08)	1.65 (2.08)	1.67 (2.25)	1.50 (1.99)	0.716
Chlorine (mEq/L)	104.09 (7.89)	104.45 (5.99)	104.89 (4.27)	103.02 (11.51)	0.091
Potassium (mEq/L)	4.15 (0.64)	4.15 (0.59)	4.14 (0.61)	4.14 (0.75)	0.971
Sodium (mEq/L)	139.13 (5.03)	139.28 (5.42)	139.61 (3.15)	138.62 (4.68)	0.251

$\bar{x}$: Average; SD: Standard deviation; g, gram; dL, deciliter; μL, units per liter; mg, milligrams; mEq, milliequivalent; L, liter; Statistical tests: One-way ANOVA and Pearson’s Chi-square; * Significant differences *p* < 0.05.

**Table 3:** Factors related to the results of the MUST scale.

MUST Scale Nutritional Risk	Low Risk *n* (%)	Medium Risk *n* (%)	High Risk *n* (%)	Total	*p*
	383 (60.8)	68 (10.8)	179 (28.41)	630	
Cause of admission ICD-11					
Circulatory and respiratory diseases	211 (67)	36 (11.4)	68 (21.6)	315 (50)	0.005 *
Neoplasms	57 (53.3)	9 (8.4)	41 (38.3)	107 (17)	
Nervous and musculoskeletal diseases and trauma	54 (65.1)	9 (10.8)	20 (24.1)	83 (13.2)	
Endocrine and digestive diseases	22 (46.8)	7 (15)	18 (38.3)	47 (7.5)	
Infectious or parasitic diseases	18 (54.6)	1 (3)	14 (42.4)	33 (5.2)	
Other diseases	21 (46.7)	6 (13.3)	18 (40)	45 (7.1)	
Gastrointestinal symptoms					
Presence of hyporexia	63 (46.3)	8 (5.9)	65 (47.8)	136 (21.6)	<0.001 *
Absence of hyporexia	320 (64.8)	60 (12.2)	114 (23.1)	494 (78.4)	
Presence of abdominal distension	108 (53.5)	21 (10.4)	73 (36.1)	202 (32.1)	0.012 *
Absence of abdominal distension	275 (64.3)	47 (11)	106 (24.8)	428 (67.9)	
Presence of diarrhea	66 (48.9)	10 (7.4)	59 (43.7)	135 (21.4)	<0.001 *
Absence of diarrhea	317 (64)	58 (11.7)	120 (24.2)	495 (78.6)	
Presence of abdominal pain	128 (54)	20 (8.4)	89 (37.6)	237 (37.6)	<0.001 *
Absence of abdominal pain	255 (64.9)	48 (12.2)	90 (22.9)	393 (62.4)	
Presence of swallowing difficulties	78 (51)	5 (3.3)	70 (45.7)	153 (24.3)	<0.001 *
Absence of swallowing difficulties	305 (63.9)	63 (13.2)	109 (22.9)	477 (75.7)	
Presence of emesis	116 (53.2)	16 (7.3)	86 (39.5)	218 (34.6)	<0.001 *
Absence of emesis	267 (64.8)	52 (12.6)	93 (22.6)	412 (65.4)	
Nutritional support route					
Oral route	342 (64.4)	64 (12.1)	125 (23.5)	531 (84.3)	<0.001 *
Enteral route	36 (46.8)	4 (5.2)	37 (48.1)	77 (12.2)	
Parenteral route	5 (22.7)	0	17 (77.3)	22 (3.5)	

Statistical tests: Pearson’s Chi-square; * Significant differences *p* < 0.05.

**Table 4:** Factors related to mortality in the ICU.

Variables	$\mathrm{Alive}\;\bar{x}$ (±SD) *n* (%)	$\mathrm{Dead}\;\bar{x}$ (±SD) *n* (%)	*p*
Nutritional risk according to MUST			
High risk	149 (26.8)	30 (41.1)	0.037 *
Medium risk	62 (11.1)	6 (8.2)	
Low risk	346 (62.1)	37 (50.7)	
Hematological parameters			
Hemoglobin (g/dL)	11.60 (2.63)	10.39 (20.06)	<0.001 *
Leukocytes (×10^3^/μL)	10.52 (17.92)	11.85 (10.52)	0.533
Lymphocytes (×10^3^/μL)	2.12 (10.39)	1.10 (0.56)	0.396
Biochemical parameters			
BUN (mg/dL)	24.32 (16.89)	35.45 (25.33)	<0.001 *
Creatinine (mg/dL)	1.55 (2.06)	2.02 (2.08)	0.068
Chlorine (mEq/L)	104.15 (6.78)	103.61 (13.21)	0.728
Potassium (mEq/L)	4.13 (0.62)	4.23 (0.77)	0.318
Sodium (mEq/L)	139.17 (4.73)	138.78 (6.86)	0.631

$\bar{x}$: Average; ±SD: Standard deviation; g, gram; dL, deciliter; μL, units per liter; mg, milligrams; mEq, milliequivalent; L, liter; Statistical test: Pearson’s Chi-square and Student’s *t*-test; * Significant differences *p* < 0.05.

**Table 5:** Multivariate logistic regression model of nutritional risk association.

Raw Estimate (Unadjusted)						Adjusted Estimate		
Variables	Low Nutritional Risk	Medium/High Nutritional Risk	OR	95% CI	*p*	OR	95% CI	*p*
Hyporexia								
No	320 (64.78)	174 (35.22)	1 (Ref.)	-	-	1 (Ref.)	-	-
Yes	63 (46.32)	73 (53.68)	0.47	0.32–0.69	0.000	1.82	1.22–2.71	0.003
Interpretation of hemoglobin levels								
Normal range	122 (65.95)	63 (34.05)	1 (Ref.)	-	-	1 (Ref.)	-	-
Polycythemia	45 (83.33)	9 (16.67)	0.39	0.18–0.84	0.017	0.38	0.17–0.84	0.017
Anemia	216 (55.24)	175 (44.76)	1.57	1.09–2.26	0.015	1.31	0.89–1.91	0.168
Nutritional support route								
Oral support	342 (64.41)	189 (35.59)	1 (Ref.)	-	-	1 (Ref.)	-	-
Enteral support	36 (46.75)	41 (53.25)	2.06	1.27–3.34	0.003	1.85	1.13–3.04	0.015
Parenteral support	5 (22.73)	17 (77.27)	6.15	2.23–16.94	0.000	5.61	2.00–15.74	0.001
**Evaluation of the associative model for nutritional risk**								
			**Reference test**					
**Diagnostic test**			**Medium/High Nutritional Risk**		**Low Nutritional Risk**		**Total**	
Positive			98		69		167	
Negative			149		314		463	
Total			247		383		630	
			**Value**		**CI (95%)**			
Sensitivity (%)			39.68		33.37		45.98	
Specificity (%)			81.98		78.00		85.96	
Validity index (%)			65.40		61.60		69.19	
Predictive value +			58.68		50.92		66.45	
Predictive value −			67.82		63.46		72.18	
Prevalence (%)			39.21		35.31		43.10	
Youden index			0.22		0.14		0.29	
Likelihood ratio +			2.20		1.69		2.87	
Likelihood ratio −			0.74		0.66		0.82	

OR: Odds Ratio; CI: Confidence Interval; Qualitative variables: absolute frequency (relative frequency); Cox and Snell’s R^2^: 0.073; Nagelkerke’s R^2^: 0.099; +: Positive; −: Negative.

## Data Availability

The data are not publicly available, due to ethical reasons indicated by the research committee of the health institution, regarding the handling and privacy of patient data.

## Supplementary Materials

The following supporting information can be downloaded at: https://www.mdpi.com/article/10.3390/jcm13051236/s1, Table S1. Hematological and biochemical variables and their measurement.
