# Supplementary material for: Factors Associated with Nutritional Risk Assessment in Critically Ill Patients Using the Malnutrition Universal Screening Tool (MUST)
Source: J Clin Med. 2024 Feb 21;13(5):1236. doi: 10.3390/jcm13051236 (PMC10931933; doi:10.3390/jcm13051236)
Supplement: Supplementary file 1 [file jcm-13-01236-s001.zip › jcm-2797839-supplementary.pdf]

Table S1. Hematological and biochemical variables and their measurement

**Hematological parameter**		
Hemoglobin	Normal range	12.3–15.3 g/dL
	Anemia	<12.3 g/dL
	Polycythemia	>15.3 g/dL
Leukocyte levels	Normal range	4.5–11 × 10^3/uL
	Leukocytosis	>11 × 10^3/uL
	Leukopenia	<4.5 × 10^3/uL
Lymphocyte levels	Normal range	1.0– 4.8 × 10^3/uL
	Lymphocytosis	>4.8 × 10^3/uL
	Lymphopenia	<1.0 × 10^3/uL
**Biochemical parameters**		
Urea nitrogen levels	Normal range	7–20 mg/dL
	Increased	>20 mg/dL
	Decreased	<7 mg/dL
Creatinine levels	Normal range	0.7–1.4 mg/dL
	Increased	>1.4 mg/dL
	Decreased	<0.7 mg/dL
Chlorine levels	Normal range	96 – 106 mEq/L
	Hyperchloremia	>106 mEq/L
	Hypochloremia	<96 mEq/L
Potassium levels	Normal kalemia	3.5 – 5.0 mEq/L
	Hyperkalemia	>5 mEq/L
	Hypokalemia	<3.5 mEq/L
Sodium levels	Normal range	135 – 145 mEq/L
	Hypernatremia	>145 mEq/L
	Hyponatremia	<135 mEq/L

Abbreviations: g, gram; dL, deciliter; uL, units per liter; mg, milligrams; mEq, milliequivalent; L, liter.
